# Posttranslational Modification of Intercellular Messenger Systems

**DOI:** 10.3389/fendo.2014.00027

**Published:** 2014-03-05

**Authors:** Sho Kakizawa, Hiroyuki Kaiya, Akiyoshi Takahashi

**Affiliations:** ^1^Department of Biological Chemistry, Graduate School of Pharmaceutical Sciences, Kyoto University, Kyoto, Japan; ^2^Department of Biochemistry, National Cerebral and Cardiovascular Center Research Institute, Suita, Japan; ^3^School of Marine Biosciences, Kitasato University, Sagamihara, Japan

**Keywords:** posttranslational modification, hormone, gaseous messenger, endocrine system, receptor

While it is estimated that the human genome comprises ~27,000 genes, the total number of proteins in the human proteome is estimated at over one million. In addition to changes at the transcriptional and mRNA levels, “posttranslational modification of proteins” increases the functional diversity of the proteome. Now, it is increasingly recognized that posttranslational modifications of proteins provide important roles in a wide range of “intercellular signaling pathways,” such as endocrine systems. For example, *n*-octanoyl modification at Ser(3) is essential for ghrelin-induced bioactivities. Moreover, gaseous messengers, such as nitric oxide and hydrogen sulfide are highly active and affect the functions of target proteins by S-nitrosylation and S-sulfhydration, respectively.

This Research Topic is aimed to assemble a series of review articles and original research papers on structural analysis or functional significance of posttranslational modification of/by intercellular messengers, including hormonal messengers and gaseous messengers, in vertebrates and invertebrates: posttranslational modification of peptide hormones such as proopiomelanocortin (1, 2), ghrelin (3–5), and hormonal receptors and effectors (6–8). Review articles on gaseous messengers such as hydrogen sulfide (9) and nitric oxide (10) are also included. The contributing papers illustrate variety and importance of biological events regulated by posttranslational modification of functional molecules, and may become major references for those working in the field of physiology and cell biology.
